# Configuration-Invariant Sound Localization Technique Using Azimuth-Frequency Representation and Convolutional Neural Networks

**DOI:** 10.3390/s20133768

**Published:** 2020-07-05

**Authors:** Chanjun Chun, Kwang Myung Jeon, Wooyeol Choi

**Affiliations:** 1 Future Infrastructure Research Center, Korea Institute of Civil Engineering and Building Technology (KICT), Goyang 10223, Korea; chanjunchun@kict.re.kr; 2 IntFlow Co., Ltd., Gwangju 61080, Korea; kmjeon@int-flow.com; 3 Department of Computer Engineering, Chosun University, Gwangju 61452, Korea

**Keywords:** azimuth-frequency representation, configuration-invariant, convolutional neural network (CNN), sound localization

## Abstract

Deep neural networks (DNNs) have achieved significant advancements in speech processing, and numerous types of DNN architectures have been proposed in the field of sound localization. When a DNN model is deployed for sound localization, a fixed input size is required. This is generally determined by the number of microphones, the fast Fourier transform size, and the frame size. if the numbers or configurations of the microphones change, the DNN model should be retrained because the size of the input features changes. in this paper, we propose a configuration-invariant sound localization technique using the azimuth-frequency representation and convolutional neural networks (CNNs). the proposed CNN model receives the azimuth-frequency representation instead of time-frequency features as the input features. the proposed model was evaluated in different environments from the microphone configuration in which it was originally trained. for evaluation, single sound source is simulated using the image method. Through the evaluations, it was confirmed that the localization performance was superior to the conventional steered response power phase transform (SRP-PHAT) and multiple signal classification (MUSIC) methods.

## 1. Introduction

The sound localization technique indicates that the location of each sound source is estimated with respect to the microphones. The time difference of arrival (TDOA) can be estimated based on the knowledge of the microphone configuration, and can be converted into the sound source location information [1]. It is an essential technique in microphone array processing and forms a fundamental part of speech enhancement, multi-channel sound separation, and distant speech recognition. Popular conventional approaches include the generalized cross correlation phase transform (GCC-PHAT) [2], the steered response power phase transform (SRP-PHAT) [3], and the multiple signal classification (MUSIC) [4], respectively. The GCC-PHAT tries to estimate the relative time delay, satisfying the maximum correlation among captured sound signals [2]. Conversely, the SRP-PHAT technique tries to generate various beams at various locations and searches for peaks in the output power signal using an arbitrarily selected resolution [3]. The MUSIC method is based on the eigenvalue decomposition of the covariance matrix captured from a microphone array [4].

Recently, numerous deep neural networks (DNNs) have been utilized to estimate the target sound source in sound localization. Takeda and Komatani estimated the TDOA through a fully connected network using eigen vector features used in MUSIC [5]. In addition, Chakrabarty and Habets performed sound localization based on convolutional neural networks (CNNs) with the use of the phase spectrum [6,7]. The convolutional recurrent neural network (CRNN) was also utilized for the estimation of the directions of arrival (DOA) of multiple sound sources [8]. By using magnitude and phase spectra, Adavanne et al. estimated both the azimuth and elevation [8]. Numerous other studies on sound localization exist. In [9], sound event detection (SED) and localization have been performed in conjunction, whereby the author described the existing methods of sound localization.

Thus far, DNN-based sound localization techniques have been primarily based on supervised learning [10]. The sound signals are recorded or simulated using the image method used for training according to the azimuth [11]. Subsequently, the DNN model is trained using spectral features between channels. This training process needs to tune the hyperparameters, and thus requires considerable time. Herein, if the numbers or configurations of the microphones change, the DNN model needs to be retrained. In that case, the hyperparameters need to be tuned again. For instance, the input of CNN in [6] is a matrix with a size of *M* x *K*, where *M* and *K* indicate the number of microphones and the number of frequency bins, respectively. If the number of microphones changes, the model need to be retrained because the input size received by the CNN does not match. Furthermore, if the distances between the microphones are changed, retraining is also necessary because the phase information that can appear in each azimuthal direction will be different.

In this paper, we propose a configuration-invariant sound localization technique, which can operate in any linear microphone array. The proposed CNN model receives the azimuth-frequency representation instead of time-frequency features as the input features. After converting the phase information to an azimuthal direction, it is utilized as a CNN input feature. We generated four microphone configurations in this study. Two microphone configurations were used for training, and the other two were used for validation and evaluation, respectively. Single sound signals were simulated using the image method [11], and different room impulse response (RIR) characteristics were used based on the use of different parameters for each microphone configuration.

This paper is organized as follows. Following this introduction, Section 2 describes the sound signal conversion method to an azimuth-frequency representation. Subsequently, Section 3 includes the overall structure of the neural network and the sound dataset. In Section 4, the performance of the proposed sound localization technique is evaluated according to the different microphone configurations. Finally, Section 5 concludes the study.

## 2. Azimuth-Frequency Representation from Sound Signal

The captured sound signals from a microphone array are represented as a delayed and attenuated version of the desired signal, s(n), such that

(1) $$\begin{array}{c} \left[\begin{array}{c} x_{1}\left(n\right)\\ \vdots \\ x_{M}\left(n\right) \end{array}\right]=\left[\begin{array}{c} a_{1}s\left(n-\tau_{1}\right)\\ \vdots \\ a_{M}s\left(n-\tau_{M}\right) \end{array}\right]+\left[\begin{array}{c} v_{1}\left(n\right)\\ \vdots \\ v_{M}\left(n\right) \end{array}\right] \end{array}$$

where $a_{i}$ is the attenuation factor, and $\tau_{i}$ is the relative time delay between the origin and *i*-th microphone. *M* indicates the number of microphones. Moreover, $v_{i}\left(n\right)$ refers to the ambient noise component recorded from the *i*-th microphone. Note that $a_{i}$ can be approximated as 1 if the distances between successive microphones are close [12]. It is assumed that the sound source is significantly farther from the microphone array. The sound source is then propagated to all microphones in parallel based on a far-field model [12].

In addition, $\tau_{i}$ can be determined by the sound speed, *c*, the spacing between the origin and the *i*-th microphone, $l_{i}$, and the sound direction, θ. Applying a *K*-point short-time Fourier transform (STFT), Equation (1) can be represented as

(2) X=dS(k)+V,

where $X^{T}=\left[X_{1}\left(k\right)\cdots X_{M}\left(k\right)\right]$ and $V^{T}=\left[V_{1}\left(k\right)\cdots V_{M}\left(k\right)\right]$. Herein, S(k) is the *k*-th spectral component of s(n), and d is a steering vector,

(3) $$d=\left[\begin{array}{c} \exp\left(-j\frac{2\pi k}{K}\frac{f_{s}}{c}l_{1}\sin\theta \right)\\ \vdots \\ \exp\left(-j\frac{2\pi k}{K}\frac{f_{s}}{c}l_{M}\sin\theta \right) \end{array}\right].$$

This equation signifies the sound source at direction, θ, and is related to the time delay. By extending this concept, we can obtain the azimuth-frequency representation, given that

(4) $$AF\left(k,\theta \right)=\frac{1}{M}\left|\sum_{i=1}^{M}\exp\left(-j\frac{2\pi k\tau_{i}\left(\theta \right)}{K}\right)X_{i}\left(k\right)\right|,$$

where $\tau_{i}\left(\theta \right)=\left(f_{s}/c\right)l_{i}\sin\theta .$ Here, log(AF(k,θ)+1) is utilized because the amount of energy varies depending on the frequency.

**Figure 1:**
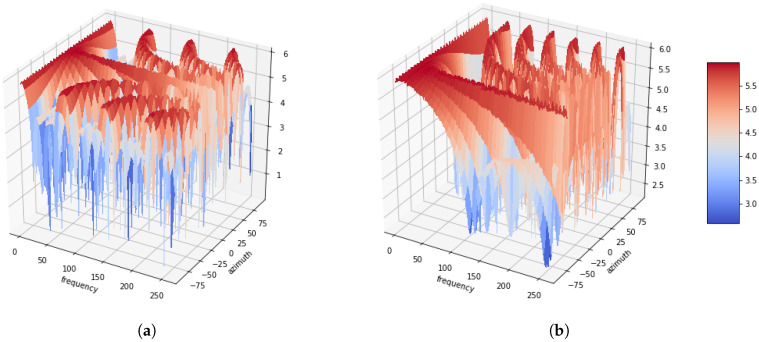

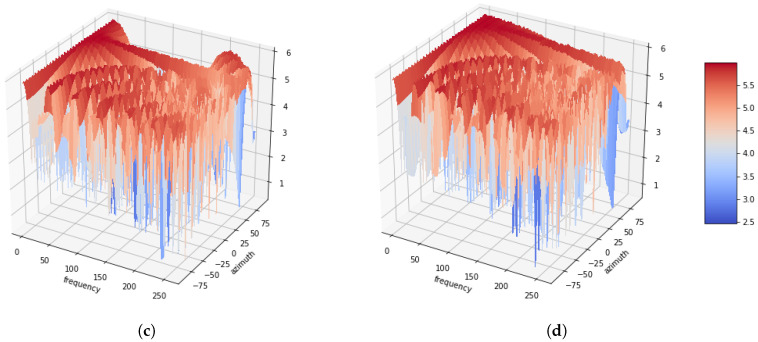
Azimuth-frequency representation of white noise depending as a function of the azimuth. (**a**) Azimuth = 0${}^{{}^{\circ}}$. (**b**) Azimuth = −60${}^{{}^{\circ}}$. (**c**) Azimuth = 45${}^{{}^{\circ}}$. (**d**) Azimuth = 90${}^{{}^{\circ}}$.

**Figure 2:**
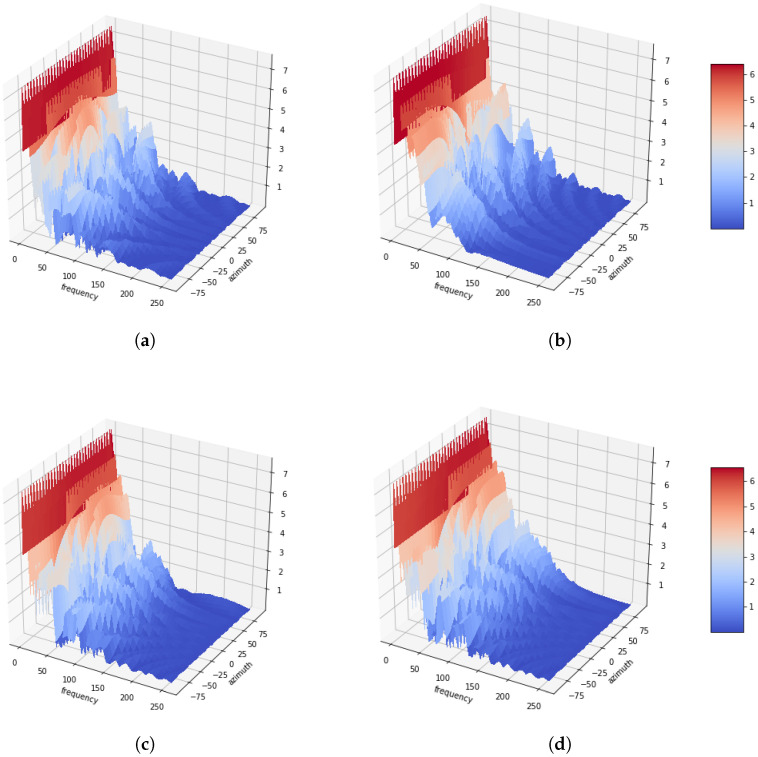
Azimuth-frequency representation of speech signal depending as a function of the azimuth. (**a**) Azimuth = 0${}^{{}^{\circ}}$. (**b**) Azimuth = −60${}^{{}^{\circ}}$. (**c**) Azimuth = 45${}^{{}^{\circ}}$. (**d**) Azimuth = 90${}^{{}^{\circ}}$.

Figure 1 illustrates the azimuth-frequency representation, whereby white noises are associated with the azimuthal angles of 0${}^{\circ }$, −60${}^{\circ }$, 45${}^{\circ }$ and 90${}^{\circ }$, respectively. Four microphones are utilized with distances between successive microphones of 5 cm. Additionally, a 512-point STFT is applied to each frame of the signals, and the azimuth is discretized in 64 steps from −90${}^{\circ }$ to 90${}^{\circ }$. As shown in the figure, it can be confirmed that the amount of energy distributed with white noise at the azimuth. Another azimuth-frequency representation using a speech signal is depicted in Figure 2. Like Figure 1, it is dominant in the azimuth direction with a speech signal. Furthermore, it can be observed that this phenomenon is more apparent in low rather than in high frequencies. This azimuth-frequency representation can be reproduced by any linear microphone configuration. A far-field model is assumed in this study, and then the time delay is only considered. However, the azimuth-frequency representation can be also generated by the attenuation term [13]. In addition, this azimuth-frequency representation can be utilized for the frequency-dependent amplitude panning to enhance the stereophonic image [14].

When a DNN model is utilized for sound localization, a fixed input size is necessary. Therefore, the model should be retrained depending on the number of microphones. Furthermore, it is also essential to retrain the model if the microphone spacing varies. The phase difference is a very important cue for sound localization. If the microphone spacing varies, the phase difference also differs. However, the azimuth-frequency representations converts this phase information into azimuth information, so it is not affected by microphone configuration.

## 3. Sound Localization using Convolutional Neural Network

This section introduces the principle of the sound localization technique using the azimuth-frequency representation. First, the synthesized data used for training a CNN model are described. Subsequently, the architecture of the convolutional neural network is presented.

### 3.1. Dataset

To train the CNN model, the Korean speech DB was utilized. This database (DB) was composed of data from four males and four females, among whom two males and two females were used for training, and one male and one female were each used for validation and evaluation, respectively. Each person contributed 40 utterances, which were sampled at 48 kHz with 16 bits. This speech DB was originally constructed for speech recognition. Therefore, it is very similar to the publicly available speech DB. To utilize this speech DB for training, the image method was employed [11]. The image method is one of the most widely utilized methods in the acoustic signal processing to simulate the synthetic room impulse responses (RIRs). This method reflects the reflection order, the room dimension, and microphone positions.

Table 1 shows the speech DB simulated through the image method. In this case, $h_{1}$ and $h_{3}$ have the same number of microphones, but the spacing between microphones is slightly different. In addition, $h_{2}$ and $h_{4}$ have two and eight microphones, respectively. The parameters related to the image method are also slightly different. Herein, $h_{1}$ and $h_{2}$ are used to generate the training DB, and $h_{3}$ is used to create the validation DB. For the generation of evaluation DB, all RIRs are utilized. The position of the sound source was placed at different locations at 10${}^{\circ }$ intervals from −90${}^{\circ }$ to 90${}^{\circ }$ whereas the distance between the sound source and the microphone was maintained, as presented in Table 1. Thus, given that one person had 40 utterances, the training DB was composed of 40 (utterances) × 4 (persons) × 19 (degrees) × 2 (RIR) utterances.

Table 2 shows how the training, validation and evaluation sets are formed through the speech DB and RIRs. It should be noted that there is no speaker overlap while the DB sets are constructed. Evaluation I can be considered as the matched convolutive reverberant environment. However, Evaluation II is the same as the reverberation environment used in the validation set, and Evaluation III can be considered as an unmatched convolutive reverberant environment.

### 3.2. Neural Network

#### 3.2.1. Architecture

**Figure 3:**
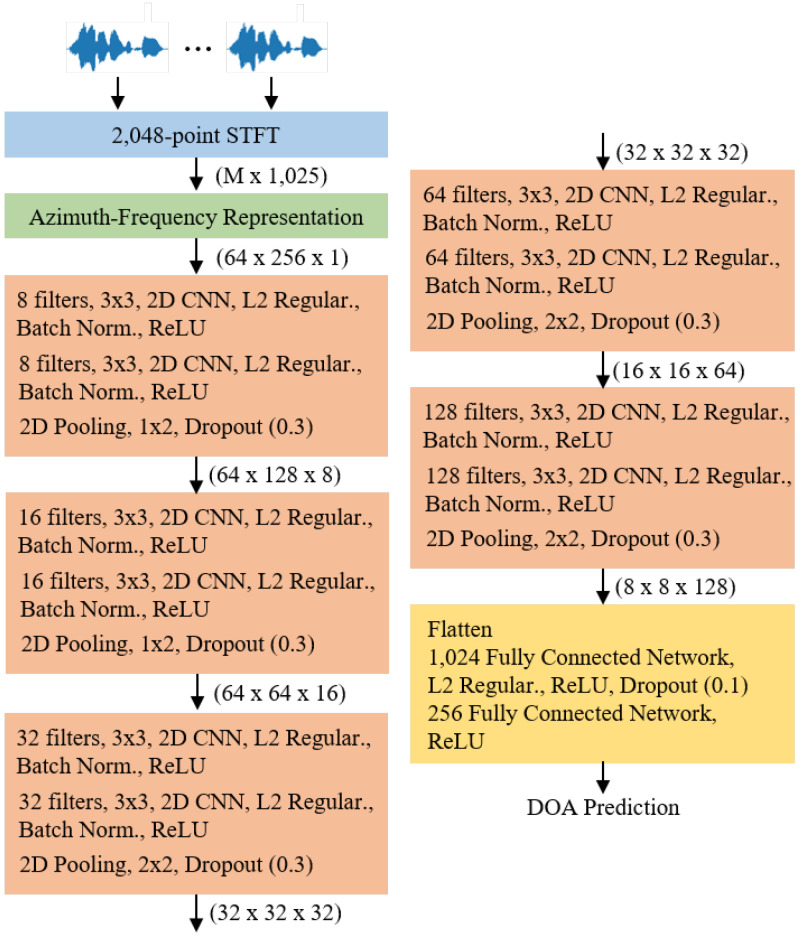
Convolutional neural network (CNN) architecture for sound localization.

Figure 3 illustrates the CNN architecture used in this study for sound localization. We created a CNN structure that was not complicated and simplified like VGG-16 [15]. First, the speech signal is converted based on 2048-point STFT. Herein, the hop length was 1024. In the azimuth-frequency representation, it was confirmed that the difference according to the azimuth direction was more distinguishable at low rather than at high frequencies. Therefore, only 256 frequency bins were used at low frequencies, with the exception of the 0th direct-current (DC) component. The azimuth was discretized in 64 steps from −90${}^{\circ }$ to 90${}^{\circ }$. All two-dimensional CNNs used kernel sizes of (3, 3) size with a (1, 1) stride [10]. L2 regularization with 0.01 was applied to all CNN layers [16]. Furthermore, after the CNN layer, batch normalization and activation functions are applied [17]. Herein, the ReLU function, which is f(x)=max(0,x), was used as the activation function [18].

A pooling layer was applied when the CNN, batch normalization, and activation layers were carried out twice. To match the size of azimuth and frequency dimension, the size of (1,2) was applied to the pooling layer at the beginning. The size of (2,2) was applied subsequently. After the pooling layer, the dropout with 0.3 was utilized [19]. In the last stage, fully connected layers were used twice after multiple CNN layers. Like the CNN layers, L2 regularization and dropout were utilized, and the corresponding parameters were 0.005 and 0.01, respectively.

#### 3.2.2. Training Method

**Figure 4:**
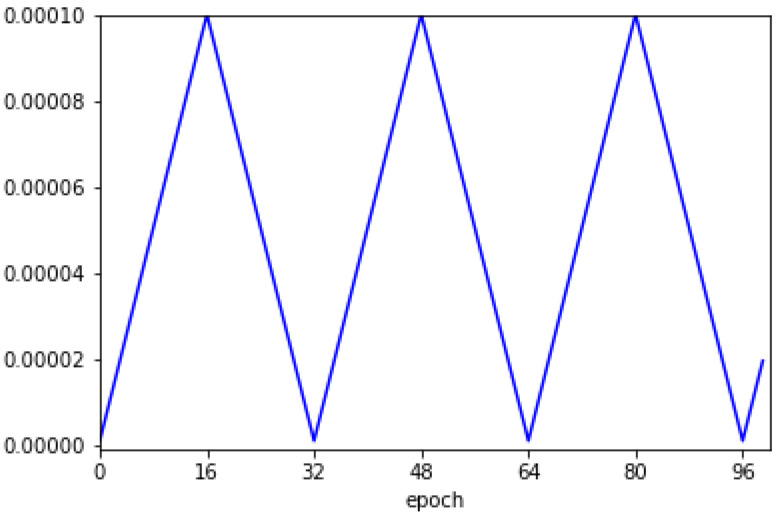
Cyclical learning rate used for training the CNN model.

**Figure 5:**
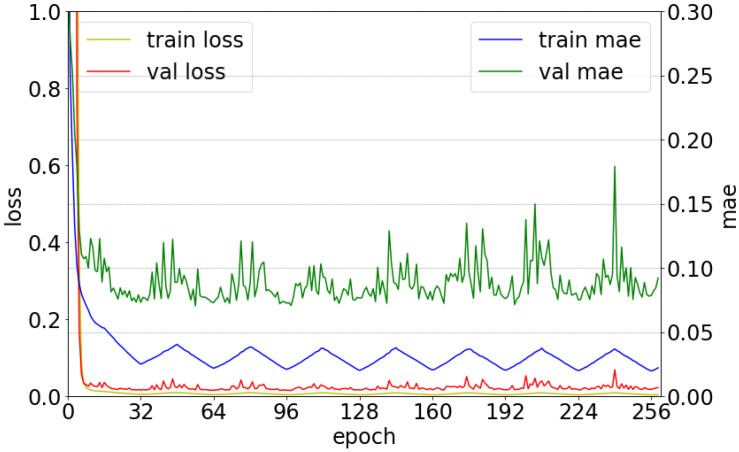
Variations of loss and mean absolute error (MAE) depending on the epochs for the training and validation sets.

The CNN model was designed based only on the consideration of only a single mixture, and the scaled azimuth degree for one sound source ranged between 0${}^{\circ }$–1${}^{\circ }$, which originally ranged between −90${}^{\circ }$–90${}^{\circ }$. The loss function was configured in the form of a mean square error (MSE) [20]. Adaptive moment estimation (Adam) was used as the optimization technique [21]. The cyclical learning rate was utilized as illustrated in Figure 4 [22]. The initial learning rate started at $10^{-6}$, and the maximum learning rate was $10^{-4}$. Herein, the step size was 16. Several ensemble techniques are used to maximize the neural network’s performance [23]. Ensemble refers to the use of not only one model that yields the best performance among several trained models, but also to the combination of results obtained from several different models. Herein, we utilized multiple snapshots of a single model during training [24]. The mean absolute error (MAE) was used as the metric, and the model with the lowest MAE was collected based on the validation set. Thus, the first model is that with the lowest MAE among the models near the 32nd epoch, and the second model is that with the lowest MAE among the models near the 64th epoch. Finally, eight models were used for sound localization. Figure 5 shows the loss value and MAE depending on the epochs for the training and validation sets. It can be confirmed that both loss and MAE follow the trend of the cyclical learning rate illustrated in Figure 4.

## 4. Performance Evaluation

To demonstrate the effectiveness of the proposed sound localization technique, an objective performance evaluation was conducted. As already described in Table 2, three evaluation sets exist. The first evaluation set is referred to as Evaluation I, and can be considered to be the matched convolutive reverberant environment as the training DB. In addition, Evaluation II is the same as the reverberation environment used in the validation set, and Evaluation III can be considered as an unmatched convolutive reverberant environment. The sound localization model in this study was used to estimate DOA on a frame-by-frame basis. Given that a single audio clip is mostly redundant (its largest proportion does not contain useful information), a DOA was estimated based on the assertion that a frame that has a specific power energy contains a speech signal. This was applied equally to MUSIC and SRP-PHAT which were used as comparison methods. If the averaged power energy was greater than 0.3, it was considered to contain speech, and it was a noise-free samples. For MUSIC and SRP-PHAT algorithms, the Python package provided in [25] was utilized.

The training and validation sets contained 780,056 and 203,876 samples, respectively. Moreover, the evaluation sets contained 359,103, 195,440, and 180,850 samples, respectively. Although the numbers of utterances were the same, the lengths of the spoken sentences were slightly different, and the number of frames included as silence frames could differ accordingly.

**Figure 6:**
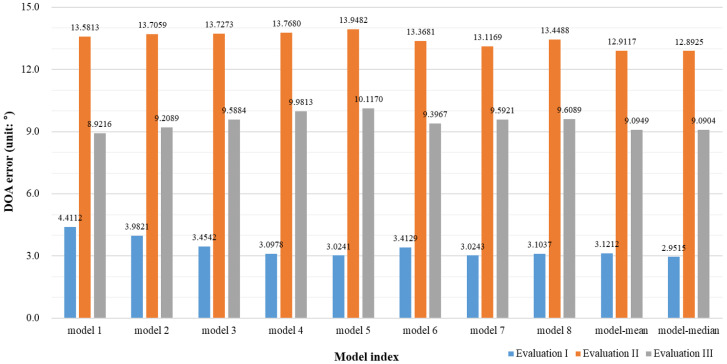
Direction of arrival (DOA) error depending on the evaluation set.

Figure 6 shows the DOA error depending on the evaluation set. First, it can be confirmed that the performance is slightly improved when all models are used together compared to the case at which each model is handled individually. Additionally, the performance was somewhat better when the median was used than when the mean was used. Depending on the evaluation set, Evaluation I yielded the lowest DOA error, and Evaluation III yielded the highest DOA. This is because Evaluation I was a matched convolutive reverberant environment with the same reverberation characteristics as those used for training. However, Evaluation II and Evaluation III were unmatched environments. Evaluation II generated the azimuth-frequency representation with a total of four microphones, while Evaluation III generated the azimuth-frequency representation with a total of eight microphones. Therefore, more accurate and sophisticated azimuth-frequency features can be generated when several microphones are used that reduce DOA errors.

Table 3 shows the DOA error depending on the sound localization technique. It is possible to confirm that the proposed CNN method contains has fewer DOA errors than those of the existing MUSIC or SRP-PHAT techniques. In the conventional method, the SRP-PHAT method yielded DOA errors smaller than that of the MUSIC method. Unlike the proposed method, both the conventional methods yielded increased DOA errors in Evaluations I and II. In the DNN approach, reverberation characteristics can be memorized during training. Thus, the matched environment yielded a small DOA error. However, the conventional method did not. Furthermore, Evaluation I yielded the highest DOA error, and Evaluation III yielded the smallest DOA error in the conventional techniques. It can be implied that the DOA error decreased as the number of microphones increased. In Evaluation I, $h_{1}$ had four microphones, but $h_{2}$ had only two microphones. Moreover, $h_{3}$ and $h_{4}$ had four and eight microphones, respectively.

## 5. Conclusions

We proposed the configuration-invariant sound localization technique using the azimuth-frequency representation. When a CNN model was utilized for sound localization, a fixed input size was required. Therefore, it was necessary to retrain the model depending on the microphone configuration. To eliminate this problem, we utilized the azimuth-frequency representation as input features, and the experiment was conducted with four microphone configurations generated by four different RIR filters. The CNN model was trained only in the first and second configurations with four microphones (spaced 3 cm apart) and two microphones (spaced 3 cm apart), respectively. The trained CNN model outperformed the existing MUSIC and SRP-PHAT methods in the same microphone configuration with the same convolutive reverberant environments. In addition, even though the microphone configuration was changed, it was confirmed that better performance was achieved without any additional retraining procedure than the existing MUSIC and SRP-PHAT methods. In the future, it is expected that the azimuth-frequency representation can be upgraded to estimate multiple sound sources and to simultaneously detect sound events.

## Figures and Tables

**Table 1:** Speech database (DB) simulated with the image method [11].

	Number of Microphones	Spacing between Microphones (m)	Distance from Source to Microphone (m)	Position of Microphone Origin (m)	Room Size (m)	Reflection Coefficient	Number of Images
$h_{1}$	4	0.03	1.5	[4, 1, 1.5]	[8, 6, 3.5]	0.7	12
$h_{2}$	2	0.03	1.2	[3.5, 2, 1.4]	[7, 5, 3.3]	0.8	15
$h_{3}$	4	0.04	1.4	[6, 1.5, 1.6]	[12, 6, 3.8]	0.85	13
$h_{4}$	8	0.03	1.8	[5, 2.5, 1.7]	[10, 8, 4.2]	0.72	15

**Table 2:** Training, validation, and evaluation sets depending on RIRs

	Speech I (Two Males, Two Females)	Speech II (One Male, One Female)	Speech III (One Male, One Female)
$h_{1}$	Training	-	Evaluation I
$h_{2}$			
$h_{3}$	-	Validation	Evaluation II
$h_{4}$	-	-	Evaluation III

**Table 3:** Direction of arrival (DOA) error depending on sound localization technique.

Units (°)	Conventional		Proposed	
	MUSIC	SRP-PHAT	Mean	Median
Evaluation I ($h_{1}$ and $h_{2}$)	30.4253	30.8505	3.1212	2.9515
Evaluation II ($h_{3}$)	31.1670	23.3249	12.9117	12.8925
Evaluation III ($h_{4}$)	21.5451	13.1966	9.0949	9.0904
